# TerraClimate, a high-resolution global dataset of monthly climate and climatic water balance from 1958–2015

**DOI:** 10.1038/sdata.2017.191

**Published:** 2018-01-09

**Authors:** John T. Abatzoglou, Solomon Z. Dobrowski, Sean A. Parks, Katherine C. Hegewisch

**Affiliations:** 1 University of Idaho, Department of Geography, 875 Perimeter Dr, Moscow, ID 83844, USA; 2 University of Montana, Department of Forest Management, Missoula, MN 59812, USA; 3 USDA Forest Service, Rocky Mountain Research Station, Aldo Leopold Wilderness Research Institute, 790 E. Beckwith Ave, Missoula, MT 59801, USA

**Keywords:** Biogeography, Hydrology, Climate and Earth system modelling

## Abstract

We present TerraClimate, a dataset of high-spatial resolution (1/24°, ~4-km) monthly climate and climatic water balance for global terrestrial surfaces from 1958–2015. TerraClimate uses climatically aided interpolation, combining high-spatial resolution climatological normals from the WorldClim dataset, with coarser resolution time varying (i.e., monthly) data from other sources to produce a monthly dataset of precipitation, maximum and minimum temperature, wind speed, vapor pressure, and solar radiation. TerraClimate additionally produces monthly surface water balance datasets using a water balance model that incorporates reference evapotranspiration, precipitation, temperature, and interpolated plant extractable soil water capacity. These data provide important inputs for ecological and hydrological studies at global scales that require high spatial resolution and time varying climate and climatic water balance data. We validated spatiotemporal aspects of TerraClimate using annual temperature, precipitation, and calculated reference evapotranspiration from station data, as well as annual runoff from streamflow gauges. TerraClimate datasets showed noted improvement in overall mean absolute error and increased spatial realism relative to coarser resolution gridded datasets.

## Background & Summary

Global environmental questions that invoke climate as a driver of a specific phenomenon require spatially and temporally consistent datasets. An extensive and growing collection of historical observed climate datasets exist including those that interpolate data from surface observations^1^, reanalysis^2^, and some combination thereof^3–5^ with each dataset offering advantages in the variables provided, temporal and spatial resolution, as well as the geographic extent and time period covered. Increased interest in applying climate data to multidisciplinary problems has prompted the development of climate datasets at more meaningful spatial scales. While time varying (monthly data spanning multiple decades) and high spatial resolution (<5-km) climate datasets have been developed at national and continental scales^6–8^, and high-spatial resolution climate normals have been developed for the globe^9–11^, we are unaware of time varying (i.e., by month and year), high-resolution climate data that covers all global land surfaces and encompasses many of the essential surface climate variables.

There is increasing recognition of the mismatch between the scale of existing climate data and the scale at which organisms experience their environment^12^. This has motivated a large body of work aimed at downscaling gridded climate fields for use in ecological and global change studies, the results of which may be sensitive to the spatial scale of data used^13^. High spatial resolution climate data is often necessary for ecological and hydrological analyses and modeling^14^. The newest version of WorldClim^15^ used station data, remotely sensed land surface temperature, and topographic covariates to produce high spatial resolution climate surfaces of temperature, precipitation, wind speed, vapor pressure, and downward shortwave flux. While WorldClim provides a complete set of variables to assess long-term monthly normals for several climate variables, it does not lend itself to temporal analysis that may be important for linking climate variability and climate impacts in ecological, agricultural, and hydrological systems.

In addition to temperature and precipitation, there has been interest in measures of climate that are more directly linked to ecosystem productivity and water resources. Water balance models integrate climate and biophysical factors to produce a set of variables that account for the concurrence of water and energy. Water balance models have been used to estimate runoff^16,17^, ecosystem productivity^18^, ecosystem distribution^19^, and ecological disturbance^20,21^. Whereas some global water balance datasets exist from simple water balance models^22–24^ or more sophisticated land-surface hydrologic models^25^, these models typically provide output at relatively coarse spatial scales (>50-km) or lack extensive time series components. Higher resolution (<5-km) country and continental scale water balance datasets have been developed^21^, yet global, time varying, high-resolution datasets do not currently exist.

This paper outlines the development of a global monthly high-resolution climate dataset from 1958–2015, herein referred to as TerraClimate, that includes the requisite variables for calculating energy-based reference potential evapotranspiration and a water balance model. We discuss the procedures for downscaling coarser resolution temporal anomalies from Climate Research Unit time series data version 4.0 (CRU Ts4.0)^1^ and the Japanese 55-year Reanalysis (JRA-55)^2^ from 1958–2015 with high-resolution climatological fields from WorldClim^11,15^ using climatic aided interpolation. Furthermore, we discuss the procedures used for developing the monthly water balance dataset for the period 1958–2015.

## Methods

Three global gridded climate datasets were used to develop TerraClimate, each offering distinct spatial or temporal qualities (Table 1).

The high-spatial resolution climatology from WorldClim version 2 (http://worldclim.org/version2) used thin-plate splines to interpolate station observations using covariates including MODIS derived land surface temperature and cloud cover, along with topographic features to develop monthly climate normal surfaces for global land surface^15^. Monthly climate normals for the 1970–2000 period were developed for maximum and minimum temperature, precipitation, solar radiation, vapor pressure and wind speed at four different spatial grains. We used the 2.5 arc-minute (1/24°) resolution to accommodate a high-spatial resolution (<5-km) product that is computationally tractable for developing longer time series.

We used the monthly diurnal temperature range (DTR) from WorldClim version 1.4 (http://worldclim.org/version1) rather than the WorldClim version 2 dataset, as the latter showed widespread biases in mid latitudes despite monthly mean temperature showing nominal bias (Supplementary Figs 1 and 2). The monthly DTR from WorldClim version 1.4 for the 1960–1990 period was further modified to account for the change in DTR between the 1960–1990 base period and 1970–2000 base period of WorldClim v2.0. This was facilitated by interpolating differences in monthly DTR for these two base periods in the CRU Ts4.0 data. Finally, monthly average maximum and minimum temperature climatologies for the 1970–2000 period were reconstructed using WorldClim v2.0 monthly mean temperatures and the DTR from WorldClim v1.4 as modified by CRU Ts4.0.

Time varying climate data were derived from two sources: (i) Climate Research Unit (CRU) time series data version 4.0 (ref. 1) (Data Citation 1), and (ii) the Japanese 55-year Reanalysis (JRA-55 (ref. 2); http://jra.kishou.go.jp/JRA-55/index_en.html). The CRU Ts4.0 data provide monthly average maximum and minimum temperature, vapor pressure, and cumulative precipitation at a 0.5° grid from 1901–2015 for global land surface and were developed based on station data. The CRU Ts4.0 data however does not cover all islands and does not include Antarctica. Furthermore, CRU Ts4.0 data assumes climatological values for pixels where there are no long-term stations within a decorrelation distance, resulting in voids of climate signals in poorly sampled land areas of the globe. The JRA-55 is the longest running full-observing-system modern reanalysis product, providing spatially and temporally complete data covering the period 1958-present. We calculated monthly average 10-m wind speed, downward surface shortwave flux, temperature, and precipitation data from 3-hourly products at a 1.25° grid. For both CRU Ts4.0 and JRA-55 datasets, we calculated monthly anomalies for all variables relative to the 31-year baseline period (1970–2000) defined by WorldClim v2.0.

Climatologically aided interpolation was used to superimpose monthly climate anomalies from CRU Ts4.0 and JRA-55 with monthly climate normals from WorldClim to estimate monthly time series from 1958–2015. Climatologically aided interpolation is a simple spatial downscaling approach that employs bilinear interpolation of temporal anomalies from a higher-temporal, lower-spatial resolution dataset to a lower-temporal, higher-spatial resolution dataset^26,27^. All anomalies were calculated relative to the 1970–2000 base period to adhere with the WorldClim climatology. Monthly average maximum temperature, minimum temperature, vapor pressure, and precipitation anomalies were prioritized from CRU Ts4.0 where these data cover land surface pixels. The CRU Ts4.0 data assigns pixels to their climatological normal when there are no stations within decorrelation distances^28^ in a given month. These represent a very small fraction of the temperature and precipitation record from 1958–2015. Rather than use a potentially artificial null climate signal such instances, we bilinearly interpolate JRA-55 anomalies to infill data voids. CRU Ts4.0 anomalies were further extrapolated to a 0.5° buffer from land masses by interpolating anomalies from the 8 nearest neighbors. Monthly anomalies from JRA-55 were used for pixels on islands located outside this buffer, as well as in Antarctica which is not covered by CRU Ts4.0. Anomalies of 10-m wind speed and downward surface shortwave flux were taken exclusively from JRA-55. Anomalies were computed as additive (i.e., departure from normal) for temperature, wind speed, and solar radiation, and multiplicative (i.e., percent of normal) for precipitation and vapor pressure. We capped monthly precipitation anomalies at 2,000% of normal to avoid unrealistic precipitation in exceptionally dry regions and months where climatological precipitation from CRU Ts4.0 or JRA-55 approaches zero. There were rare occurrences (~0.1% of pixels) where monthly mean maximum temperature was less than 0.5 °C higher than minimum temperature due to our method independently accounting for anomalies in maximum and minimum temperature. In such cases, we set the monthly minimum temperature equal to 0.5 °C below the monthly maximum temperature for internal consistency.

Monthly reference evapotranspiration (ET_0_) was calculated using the Penman Montieth approach^29^. ET_0_ has been argued to be a more appropriate measure of potential water loss over purely temperature based metrics for estimating potential evapotranspiration as it uses an energy balance approach. ET_0_ assumes a reference grass surface across space, but can yield biases where potential vegetation water use departs substantially from this assumption. Following prior studies, we modified ET_0_ to account for reduced surface water flux when snow cover exists or prior to the onset of the growing season and active transpiration using an empirical relationship with temperature that accounts for precipitation phase changes^16,30^.

A one-dimensional modified Thornthwaite-Mather climatic water-balance model (WBM)^22,31^ was used to calculate monthly water balance from 1958–2015. The WBM is a single bucket model applied consistently across global land surfaces that operates on a monthly time step and considers the interplay between precipitation, ET_0_, as well as soil and snowpack water storage. The WBM accounting scheme considers runoff as the excess of liquid water supply (precipitation and snowmelt) used by monthly ET_0_ and soil moisture recharge. Soil water is extracted during months where ET_0_ exceeds liquid water supply, with the extraction efficiency of soil water declining exponentially with the ratio of soil water to extractable soil water capacity. Under such conditions, actual evapotranspiration is counted as the liquid water supply plus the soil water utilized and climatic water deficit is the difference between ET_0_ and actual evapotranspiration. The WBM requires data on the plant extractable soil water capacity. We used extractable soil water storage capacity data at a 0.5° grid from Wang-Erlandsson *et al.*^32^ that were derived from estimates of satellite based evaporation, ET_0_, and precipitation. Wang-Erlandsson *et al.*^32^ provided estimates for varying return intervals. We used the 2-year period given our use of ET_0_, as it best matched with evapotranspiration variability in grassland biomes, corresponding with our use of a reference grass surface in the WBM. To adhere to the spatial resolution of TerraClimate, we first extrapolated data using a 0.5° buffer from existing data using the 8 nearest neighbors, and then bilinearly interpolated plant extractable soil water from its native 0.5° grid. We set a lower bound on plant extractable soil water of 10 mm and set a default value of 50 mm for places void of data.

We use the WBM outlined by Dobrowski *et al.*^31^, with two modifications to better account for runoff and snowpack dynamics on a global scale. First, we use an empirical temperature-based transformation for precipitation phase to determine the proportion of precipitation falling as liquid, as well as the fraction of snowpack melted each month^30^. Although snowmelt is a more complicated process that could be improved by incorporating shortwave fluxes and snowpack ripeness, temperature based indices have been shown to be sufficient for macroscale monthly water balance calculations^33^. Secondly, we allow for 5% of the cumulative rainfall and snowmelt each month to occur as direct runoff irrespective of soil conditions^17^. The resultant datasets from the WBM are monthly total runoff (Q), climatic water deficit, actual evapotranspiration, as well as snow water equivalent and soil moisture at the end of each month.

## Data Records

TerraClimate is available to the public through an unrestricted data repository hosted by the University of Idaho’s Northwest Knowledge Network (Data Citation 2). The full list of TerraClimate datasets are provided in Table 2. Files were created separately for each variable and year in NetCDF format following the Climate and Forecast metadata standards. A compressed archive of NetCDF files for the period of record covering all variables can be accessed at http://doi.org/10.7923/G43J3B0R. However, due to the sheer size of the data once uncompressed (~800 GB), we provide access to individual annual files for each variable at https://climate.northwestknowledge.net/TERRACLIMATE/. Temporal extensions to TerraClimate will be updated periodically as additional years of data become available.

## Technical Validation

We conducted a validation of the TerraClimate product for monthly temperature and precipitation using station-based data obtained from the Global Historical Climatology Network (GHCN) database^34^. We examined time series of temperature data that have been corrected for climate inhomogeneities that arise due to non-climatic artifacts, such as station relocation or observational practice, from the GHCNv3 dataset; whereas we used monthly precipitation data from the GHCNv2 dataset. We constrained our validation to GHCN stations that had complete monthly data for at least 30 calendar years from 1958–2015 resulting in a total of 3,230 stations for temperature and 6,102 stations for precipitation. As a caveat, we note that many of these stations were used in both the WorldClim and CRU datasets making the comparison not completely independent.

Data from TerraClimate were extracted at grid cells co-located with individual stations. We calculated linear validation statistics of Pearsons’ correlation coefficients, mean absolute error (MAE), and bias for each station. We constrained our validation to calendar year summaries rather than individual months to avoid artificially inflating the correlation by incorporating the seasonal cycle. To demonstrate potential added value from TerraClimate, we compare our validation statistics to CRU Ts4.0, acknowledging that by using CRU Ts4.0 in climatically aided interpolation, we should expect similar results for temporal variability.

**Figure 1:**
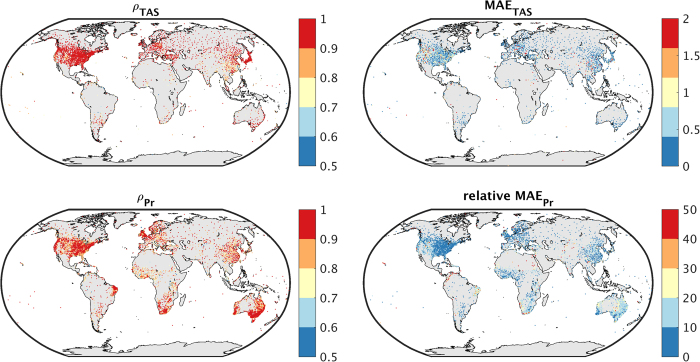
Validation of TerraClimate temperature and precipitation time series using GHCN stations. (**a**) Pearsons’ correlation coefficient (ρ) and (**b**) mean absolute error (MAE, units of °C) between GHCN stations and co-located pixels from TerraClimate for time series of annual mean temperature (TAS) from 1958–2015. (**c**,**d**) Show the correlation and relative mean absolute error (units of % of mean annual Pr) for calendar year accumulated precipitation (Pr). Statistics are reported for GHCN stations with at least 30 years of complete data.

The TerraClimate dataset largely captured interannual variability in mean annual temperature across GHCN stations with a median Pearson’s correlation coefficient (ρ) of 0.95, with ρ>0.9 for approximately 80% of the stations (Fig. 1). Most of the subpar correlations (ρ<0.8) were found equatorward of 30° latitude, where interannual variability in mean temperature is lesser in magnitude. Similarly, TerraClimate precipitation fields captured interannual variability observed at GHCN stations globally with a median correlation of ρ=0.90. Subpar correlations for annual precipitation were often found adjacent to stations with high correlation suggesting that interannual precipitation variability can vary across relatively short spatial scales, particularly where convective or orographic features are involved that influence the spatial variability of precipitation anomalies at scales finer than those resolved by CRU Ts4.0. Alternatively, subpar correlations may also derive from potential inhomogeneities or errors in station-based precipitation records.

Correlation statistics were nearly identical to those found using the CRU Ts4.0 dataset, as expected, given its use in the development of the TerraClimate dataset (Table 3). However, the MAE was substantially improved in the TerraClimate dataset, a consequence of bias correcting the data using the WorldClimv2 data. For mean temperature, the median MAE fell from 0.53 °C for CRU to 0.32 °C for TerraClimate, whereas for precipitation the median MAE fell from 11% to 9%. We also note that unaccounted-for error and associated bias inherent in comparing gridded climate data to station data is a function of mismatches in the scale of the data being compared. This is particularly apparent in regions of complex terrain where the elevation of a station may differ substantially from the average elevation of a co-located grid cell. For example, approximately 40% of the variance in the mean annual temperature bias was linearly explained by biases in elevation (i.e., grid cell elevation minus station elevation).

Given the potential fidelity problems when validating TerraClimate with GHCN stations, we perform a complementary validation of temperature and precipitation data using a network of 2,100 automated climate stations located across mountains of the western United States from the Snowpack Telemetry (SNOTEL) and Remote Automated Weather Stations (RAWS) networks. Due to suspected inhomogeneities with minimum temperature records from SNOTEL stations arising from instrumentation changes^35^, we used a set of homogeneized daily temperature data for both RAWS and SNOTEL stations^36^. Additionally, we used daily records of accumulated precipitation from a more limited set of 513 SNOTEL stations that were quality controlled^37^. Station-years where more than 5% of daily observations were missing were omitted from the validation. Due to the short period of record for most stations, we used a more liberal criterion of at least 10 years of complete data to compute interannual correlations, resulting in 1,292 stations for temperature and 503 stations for precipitation.

**Figure 2:**
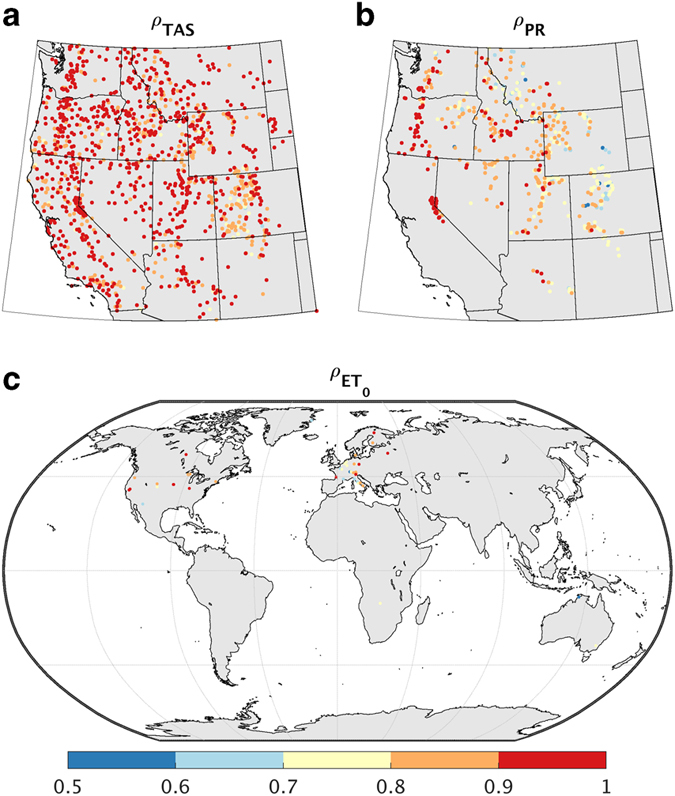
Validation of TerraClimate temperature, precipitation, and reference evapotranspiration time series. Pearsons’ correlation coefficient (ρ) with data from co-located pixels from TerraClimate and (**a**) annual mean temperature (TAS) from 1980–2015 from homogenized RAWS and SNOTEL stations, (**b**) annual mean precipitation (PR) from quality controlled SNOTEL stations, and (**c**) annual mean reference evapotranspiration (ET_0_) from FLUXNET stations from 1994–2014. Statistics are only reported for stations that had at least 10 years of complete data.

TerraClimate largely captured interannual variability in mean annual temperature across the Snotel and Raws network with a median Pearson’s correlation coefficient (ρ) of 0.90 (Fig. 2a). Interannual correlations between calendar year precipitation from TerraClimate and recorded precipitation at SNOTEL sites was also lower than seen for neighboring GHCN stations across the western US with a median ρ of 0.79 (Fig. 2b), with a dry bias (−30%) across interior mountains where correlations were generally lower. The lower correlations compared with GHCN could be a function of station quality control measures, but also factors that influence temperature and precipitation variability in mountainous areas such as inversions, snow-albedo feedbacks, and orographic precipitation ratios. In addition, the incongruence of the scales of gridded data and in-situ measurements are heightened in regions of complex terrain thus leading to an additional challenge in validation efforts. Validation statistics using CRU data showed generally similar values for correlation as those for TerraClimate, but with CRU showing slightly larger errors as noted with MAE (Table 3).

Comparisons of interannual variability in annual ET_0_ from TerraClimate were made using a global network of FLUXNET stations^38^. Hourly and bi-hourly observations of temperature, humidity, wind speed, and solar radiation from these stations were used to calculate ET_0_. Observational data were aggregated to monthly temporal scales to ensure that we used the same procedures for calculating ET_0_ as TerraClimate. Interannual correlations were computed for stations that had at least 10 years of complete data, missing no more than 10% of data in any calendar year.

Pearson’s correlations of annual ET_0_ from TerraClimate showed a median ρ of 0.77 over the 50 FLUXNET stations (Fig. 2c), with a MAE of approximately 57 mm (8.5%). The spatial correlation between observed annual mean ET_0_ and ET_0_ as modeled by TerraClimate was ρ=0.98 highlighting that the spatial pattern was well captured. A slight overall bias (median +8%) in TerraClimate ET_0_ was seen relative to ET_0_ calculated at FLUXNET sites, which may arise due to the non-standard heights relative to canopies measured at FLUXNET sites. Overall validation statistics were slightly better with TerraClimate than those seen for CRU (Table 3). In addition, less of the spatial variability in climatological ET_0_ was captured with CRU (ρ=0.94).

To demonstrate potential utility of the water balance component of the dataset, we compared interannual variability in water-year runoff from our water balance model (WBM) to streamflow from 587 pristine river basins that are part of the Climate Sensitive Stations Dataset from the Global Runoff Data Centre (The Global Runoff Data Centre, 56068 Koblenz, Germany, 2013). We considered water-year (Oct-Sep) rather than calendar year comparisons to account for the contributions of snowmelt runoff from watersheds in the northern hemisphere. These stations were selected based on having nominal water extraction or diversions, consistency of data records, at least 30 complete water-years of data from 1958–2015, and existing watershed boundary data. We extracted raster based runoff from the WBM for each gauge by delineating pixels that occurred within the upstream area of the respective watershed. The identical WBM was also run using CRU Ts4.0 datasets for comparative purposes.

**Figure 3:**
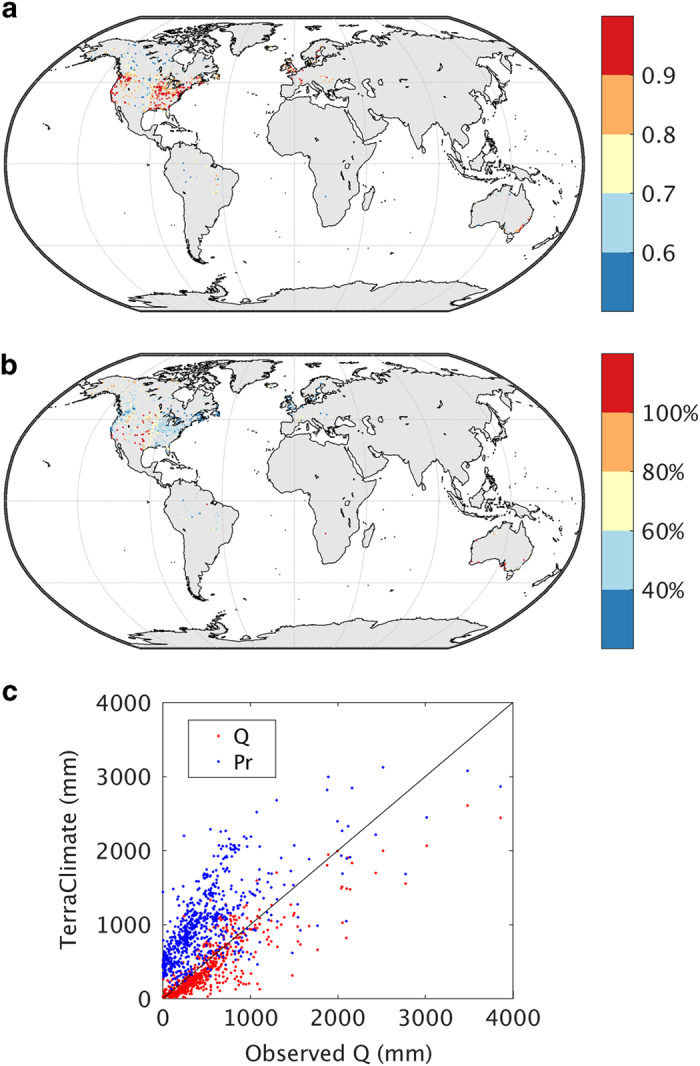
Validation of TerraClimate runoff data. Validation statistics of TerraClimate estimated runoff from the WBM showing (**a**) Pearson’s correlation coefficient, and (**b**) mean absolute error (mm) for annual water-year (Oct-Sep) runoff (Q) for 587 streamflow from BGDC’s Climate Sensitive Stations Dataset covering at least 30 complete years from 1958–2015. (**c**) Shows a scatterplot of observed annual mean Q from streamflow stations versus TerraClimate estimated Q and water-year accumulated precipitation (P). The 1:1 line is shown for reference.

Pearsons’ correlation coefficients were calculated between water-year cumulative runoff (Q) simulated by the WBM and observations. Streamflow data were scaled to units of mm per year by accounting for the size of the upstream contributing watershed. We considered temporal correlations to assess interannual variability, as well as spatial correlations to assess the performance of the dataset to capture the spatial variability in annual Q. Interannual variability in estimated runoff was reasonably captured by the WBM, with a median ρ=0.80 (Fig. 3a). The WBM captured over 70% of the interannual variability in Q of the mid-latitude basins in the United States, Europe and southern Australia. Lower explained variance was seen for parts of the tropics, boreal systems, and across the Great Plains of North America. Overall, the WBM exhibited a tendency for less runoff than was observed with a mean bias of −24%. The median relative MAE of water-year runoff was 36% (101 mm; Fig. 3b). However, MAE exceeded 100% for 6% of the watersheds, predominantly arid watersheds across the Great Plains and Australia where annual mean Q<25 mm. The spatial correlation between observed and simulated annual mean Q was ρ=0.87, suggesting that the patterns were well represented by the WBM (Fig. 3c). By comparison, these validation results were improvements over the CRU Ts4.0 dataset using the same WBM framework in terms of the median interannual correlation (ρ=0.765), relative MAE (42%, 118 mm), and the spatial correlation (ρ=0.84).

Water balance models can be biased due to several factors. Errors can arise through inaccurate estimates of climate forcings, by not accounting for vegetation dynamics within a watershed, and by the overall simplicity of the single bucket model approach which does not account for subsurface routing of water. Notably, by using ET_0_, we assume a constant reference vegetation type that may substantially deviate from observations. Additionally, plant water soil storage capacity likely deviates at scales finer than those mapped here, which could contribute to uncertainty. We conducted an additional validation analysis by comparing cumulative water-year precipitation data to observed Q for each watershed. The WBM runoff data explained substantially more variance of the interannual variability in Q than using precipitation data alone (median ρ=0.68). Observed mean runoff exceeded estimated mean annual precipitation for 30 of the 587 watersheds, further illustrating the challenges of interpolating estimates of precipitation data, particularly in mountainous watersheds as highlighted by previous studies^15,39^. Underestimates of runoff and precipitation in some mountain watersheds highlights the need for continued improvement of mapping climate at fine spatial scales globally^10,15^ and investment in observational systems in historically data sparse regions such as mountains.

**Figure 4:**
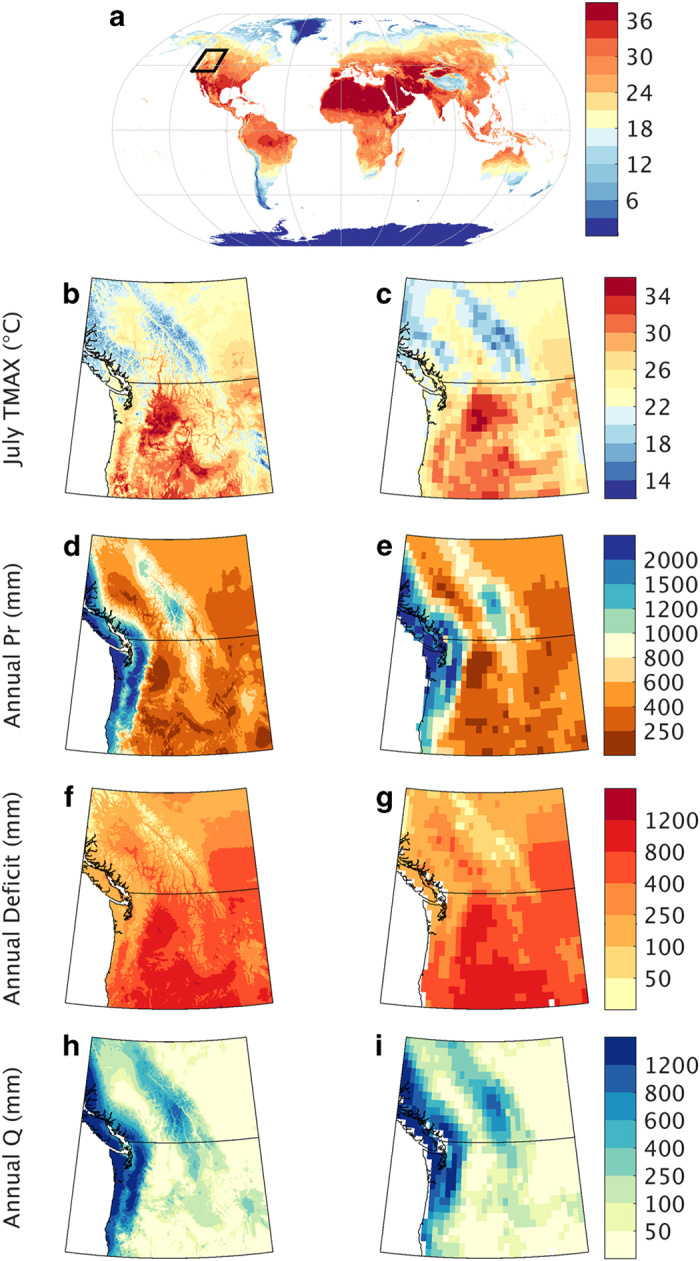
Illustration of added value from the TerraClimate dataset. Top panel shows July 2015 monthly average maximum temperature (Tmax) from TerraClimate with the inset highlighting the region for (**a**–**h**). Comparison of (**a**,**b**) July 2015 Tmax, (**c**,**d**) 2015 calendar year accumulated precipitation, (**e**,**f**) 2015 calendar year climatic water deficit, and (**g**,**h**) 2015 runoff between the TerraClimate dataset (left) and CRU Ts4.0 dataset (right).

We illustrate some of the value-added information from the TerraClimate dataset compared to CRU Ts4.0 for a portion of northwestern North America in 2015 (Fig. 4). Specifically, we highlight the spatial detail added by the TerraClimate dataset that is absent in CRU across a region with substantial geographic gradients in energy and moisture that are apparent in July 2015 average maximum temperature (Fig. 4b,c), and 2015 annual precipitation totals (Fig. 4d,e). Integrating climate metrics through the WBM improves the biophysical relevance of the gridded products for use in global-change and ecological studies, as can be seen by the additional detail in TerraClimate for annual climatic water deficit (Fig. 4f,g), and annual Q in 2015 (Fig. 4h,i).

The use of climate data has increased in the past decade due to increasing availability of data, broadening of the disciplines that use such data, and increased interest in assessing local impacts from climate change. The potential applications of TerraClimate span a variety of spatial scales from users wanting local climate and water balance time series in regions void of easily accessible or sufficiently long station-based data, to users conducting broader spatial analyses to better understand and refine regional and global climate impacts to water resources and agriculture^40^. In addition, TerraClimate has many potential uses in ecology, macroecology, and global change science. For example, water balance data are increasingly used in explaining and predicting patterns of wildfire activity^20,41,42^, vegetation distribution^19,43^ and structure^44^, climate velocity^31^, and drought induced tree mortality^45^.

## Usage Notes

Climatological data for several voxels from the WorldClim dataset near perennial sea ice or glaciers primarily in Antarctica and Greenland had exceptionally large, and likely unrealistic, vapor pressure deficits (>0.5 kPa). These voxels were primarily located near the coastline and had nominal differences in diurnal temperature range (<2 °C). Moreover, we caution users that output from the water balance model, as well as estimated ET_0_, are likely suspect in perennially glaciated landscapes including Greenland and Antarctica. Secondly, there are a few voxels near the South Pole and in the Sahara Desert where climatological 1970–2000 precipitation from WorldClim v2.0 is zero. The use of multiplicative anomalies in climatologically aided interpolation sets all values to zero, which may be suspect.

**Figure 5:**
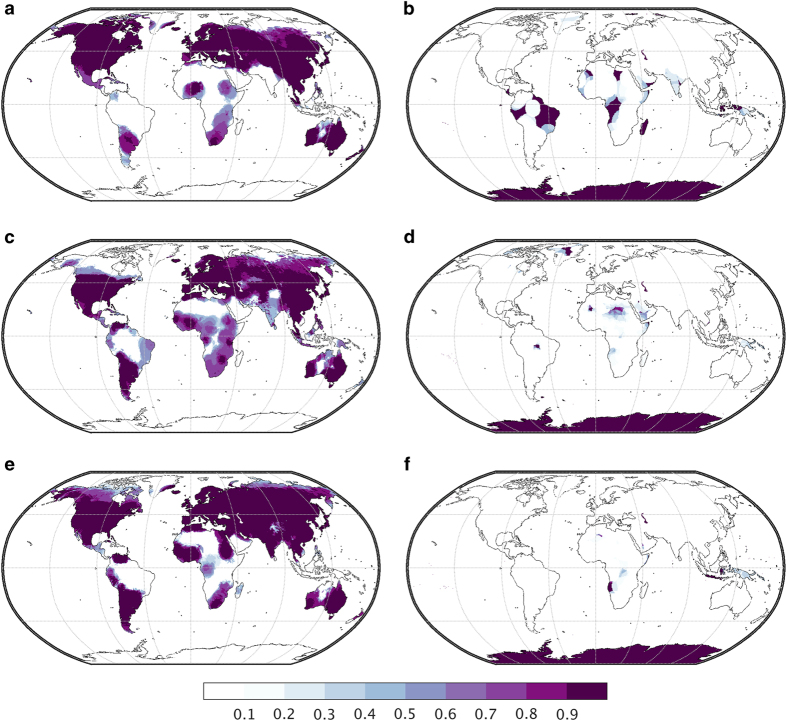
Coverage of CRU Ts4.0 station influence used in TerraClimate anomalies. Proportion of monthly data with (left) at least 8 and (right) 0 stations from CRU Ts4.0 contributing to anomalies from 1958–2015. (**a**,**b**) Show coverage for mean temperature, used in TerraClimate maximum and minimum temperature, (**c**,**d**) show coverage for precipitation, and (**e**,**f**) show coverage for vapor pressure. TerraClimate uses anomalies from JRA-55 rather than assume climatological averages from CRU for voxels where 0 stations contribute.

Uncertainty in observational gridded climate datasets that use station based data can arise from a variety of sources ranging from procedural choices for interpolating and extrapolating data spatially, to changes in data availability through time, to climate inhomogeneities that lead to structural uncertainty in climate datasets^46,47^. TerraClimate uses three different datasets, each of which contain their own sources of uncertainty. We provide a measure of uncertainty in TerraClimate data using estimates of the number of stations that contribute to anomaly fields from CRU Ts4.0 based on decorrelation distances^28^. CRU Ts4.0 provides estimates of the number of stations that contribute to estimates of monthly anomalies at the native scale of the data and provides these data for temperature, precipitation, and vapor pressure fields. The number of stations (between 0 and 8) contributing to monthly CRU Ts4.0 precipitation, mean temperature, and vapor pressure fields were interpolated to the TerraClimate grid and provided as ancillary data for precipitation, monthly maximum and minimum temperature, and vapor pressure. Anomalies from JRA-55 were used for voxels where zero stations contributed to the anomaly fields in CRU Ts4.0 data. The fraction of months from 1958–2015 with 8 contributing stations (maximum number of stations influencing) and 0 contributing stations (anomalies strictly derived from JRA-55) from CRU Ts4.0 for temperature, precipitation, and vapor pressure is shown in Fig. 5. These ancillary data are provided to help users of TerraClimate identify the robustness of interannual variability in data for specific geographic locations, as well as the data source of the interannual variability used in TerraClimate for these four variables. For example, the low fraction of months with high station density from CRU may limit the robustness of TerraClimate across regions such as Africa and South America, where temporal anomalies instead rely more heavily on JRA-55. Ancillary data are not provided for downward shortwave radiation or 10-m winds which exclusively use JRA-55 anomalies, or for ET_0_ and the water balance variables which use combinations of the essential climate variables.

Long-term trends in primary climatological fields of TerraClimate are preserved from their parent datasets in JRA-55 and CRU Ts4.0 and hence TerraClimate should not be used to provide an independent estimate of trends. Interannual variability in primary climate variables from TerraClimate will not incorporate spatial variability in anomalies at scales finer than their parent datasets, including those associated with orographic precipitation ratios, inversions, and near coastal maritime influences. Likewise, while WorldClim data incorporated into TerraClimate allows for a high-resolution dataset, such approaches may not adequately resolve microclimate features, particularly in complex terrain or heterogeneous land-cover. Some approaches have been used to better resolve microclimate features to finer spatial scales^48^, yet such relationships have been typically applied to regional domains.

## Additional information

**How to cite this article:** Abatzoglou, J. T. *et al.* TerraClimate, a high-resolution global dataset of monthly climate and climatic water balance from 1958–2015. *Sci. Data* 5:170191 doi: 10.1038/sdata.2017.191(2018).

**Publisher’s note:** Springer Nature remains neutral with regard to jurisdictional claims in published maps and institutional affiliations.

## Supplementary Material



Supplementary Figure S1

Supplementary Figure S2

## Figures and Tables

**Table 1:** Datasets used in producing the TerraClimate dataset.

**Dataset**	**Spatial Resolution Used**	**Temporal Resolution Used**	**Variables**
WorldClim v2^15^	1/24°	Monthly, 1970–2000 normals	Tas, vap, pr, srad, ws
WorldClim v1.4^11^	1/24°	Monthly, 1960–1990 normals	Tmax, Tmin, Tas
CRU Ts4.0^1^	0.5°	Monthly, 1958–2015	Tmax, Tmin, vap, pr, Tas
JRA-55^2^	1.25°	Monthly, 1958–2015	Tas, vap, pr, srad, ws
Root zone storage capacity^32^	0.5°	Time invariant	Water storage capacity
Variable abbreviations listed include average monthly maximum (Tmax), minimum (Tmin) and mean temperature (Tas), vapor pressure (vap), wind speed (ws), downward shortwave flux at the surface (srad), and accumulated monthly precipitation (pr). Note that the WorldClim datasets provide only monthly climate normals averaged over a period of record.			

**Table 2:** Datasets provided by TerraClimate.

**Dataset**	**Variables**
EssentialClimateVariables	2-m Maximum Temperature, 2-m Minimum Temperature, 2-m Vapor Pressure, 10-m Wind Speed, Downward Solar Radiation Flux at the Surface, Accumulated Precipitation
ET_0_	Reference Evapotranspiration
Water Balance Variables	Runoff, Actual Evapotranspiration, Climate Water Deficit, Soil Moisture, Snow Water Equivalent
Datasets are monthly and time varying from 1958–2015 and produced at a 1/24° spatial resolution.	

**Table 3:** Comparison of validation statistics for TerraClimate and CRU Ts4.0 for annual mean temperature (Tas), cumulative precipitation (Pr), reference evapotranspiration (ET0), and cumulative streamflow (Q).

**Variable**	**# Stations**	**Network**	**TerraClimate**		**CRU Ts4.0**	
			**ρ**	**MAE**	**ρ**	**MAE**
Tas	3,230	GHCN	0.95	0.32 °C	0.95	0.53 °C
Pr	6,102	GHCN	0.90	9.1% (62.9 mm)	0.89	11% (76 mm)
Tas	1,292	SNOTEL+RAWS	0.90	0.84 °C	0.90	1.21 °C
Pr	503	SNOTEL	0.78	30% (251 mm)	0.78	41% (316 mm)
ET_0_	50	FLUXNET	0.77	8.3% (57 mm)	0.76	8.5% (59 mm)
Q	587	GRDC	0.80	36.0% (101 mm)	0.76	42.0% (118 mm)
The median value of validation statistics for Pearsons’ correlation coefficient (ρ) and mean absolute error (MAE) are reported. Relative MAE is reported for Pr, ETo, and Q with absolute MAE shown in parentheses.						
