# Supplementary material for: TerraClimate, a high-resolution global dataset of monthly climate and climatic water balance from 1958–2015
Source: Sci Data. 2018 Jan 9;5:170191. doi: 10.1038/sdata.2017.191 (PMC5759372; doi:10.1038/sdata.2017.191)
Supplement: Supplementary Figure S1 [file sdata2017191-s2.pdf]

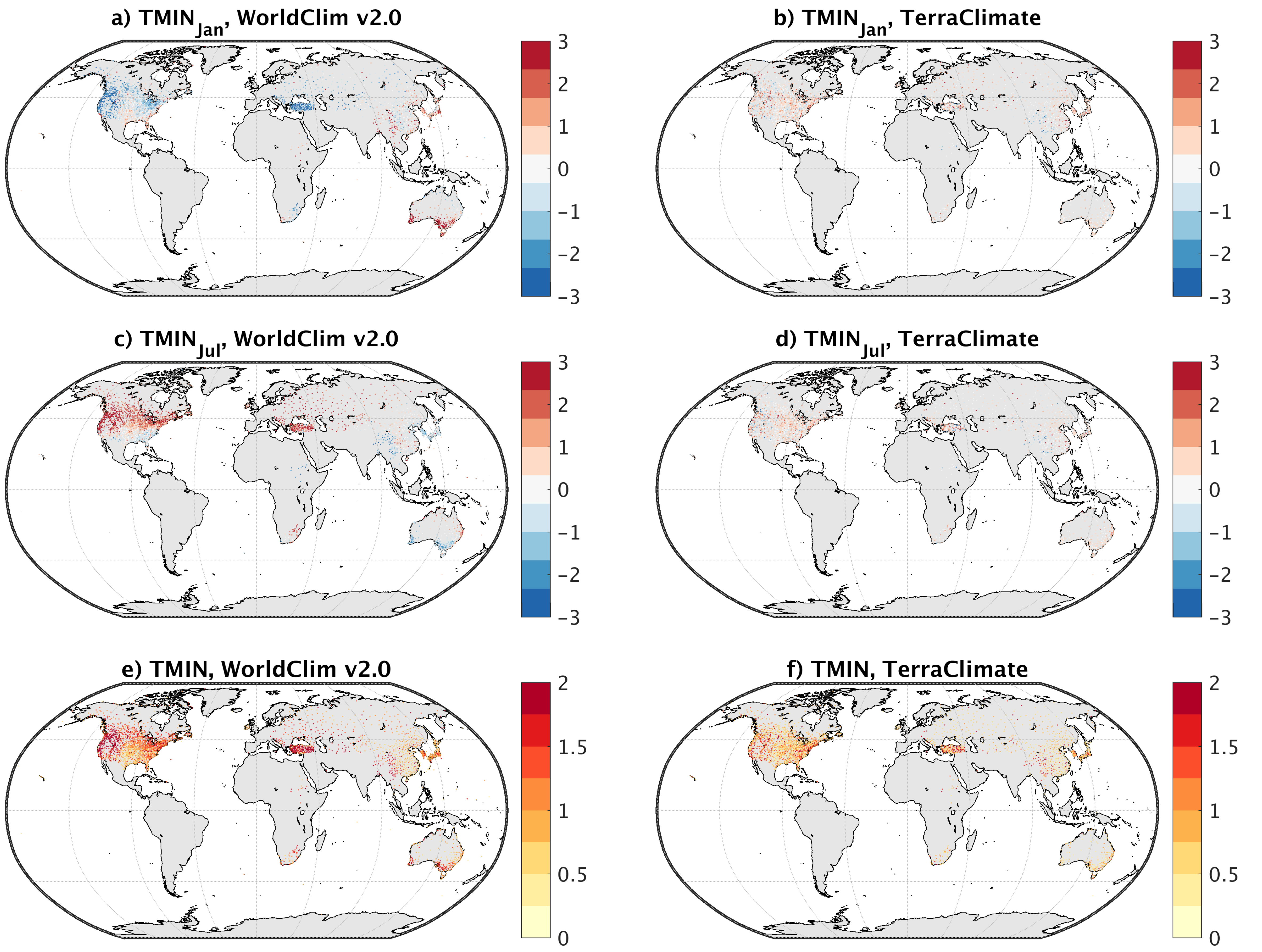
a) TMIN Jan, WorldClim v2.0
b) TMIN Jan, TerraClimate
c) TMIN Jul, WorldClim v2.0
d) TMIN Jul, TerraClimate
e) TMIN, WorldClim v2.0
f) TMIN, TerraClimate
3
2
1
0
−1
−2
−3
2
1.5
1
0.5
0
