# Supplementary material for: TerraClimate, a high-resolution global dataset of monthly climate and climatic water balance from 1958–2015
Source: Sci Data. 2018 Jan 9;5:170191. doi: 10.1038/sdata.2017.191 (PMC5759372; doi:10.1038/sdata.2017.191)
Supplement: Supplementary Figure S2 [file sdata2017191-s3.pdf]

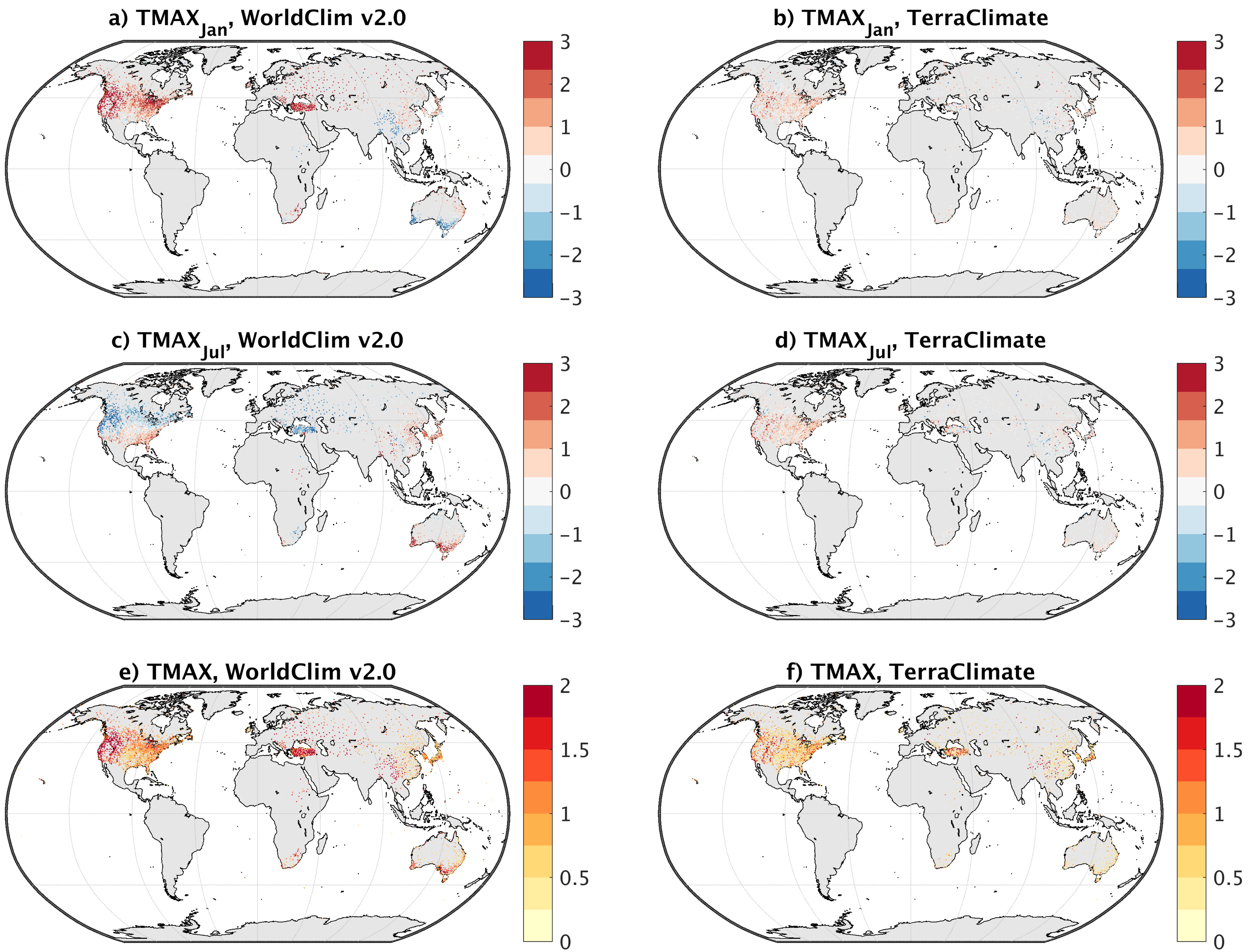

a) TMAX_Jan, WorldClim v2.0
b) TMAX_Jan, TerraClimate
c) TMAX_Jul, WorldClim v2.0
d) TMAX_Jul, TerraClimate
e) TMAX, WorldClim v2.0
f) TMAX, TerraClimate
3
2
1
0
−1
−2
−3
2
1.5
1
0.5
0
